# Remimazolam Anesthesia for Modified Electroconvulsive Therapy Mitigates Postoperative Agitation

**DOI:** 10.7759/cureus.71037

**Published:** 2024-10-07

**Authors:** Shinako Shimono, Tomoharu Shakuo, Aya Yamamura, Megumi Hashimoto, Rikuo Masuda, Kenji Shida

**Affiliations:** 1 Perioperative Medicine, Division of Anesthesiology, Showa University School of Dentistry, Tokyo, JPN; 2 Anesthesiology, Showa University Northern Yokohama Hospital, Yokohama, JPN

**Keywords:** anesthesiology, benzodiazepines, depression, modified-electroconvulsive therapy, postoperative agitation, propofol, remimazolam

## Abstract

Postoperative complications, such as immediate postoperative blood pressure elevation, agitation, and delirium, have been associated with modified electroconvulsive therapy (mECT). Remimazolam may reduce postoperative delirium; however, there are no reports of its use in mECT. Herein, we present a case of effective convulsions and calm arousal with remimazolam in a patient with a history of postoperative agitation. The patient was a 45-year-old man who was diagnosed with severe depression and psychotic symptoms and was treated with electroconvulsive therapy (ECT). Owing to previous episodes of agitation upon awakening, remimazolam and suxamethonium were administered, and mECT was performed under general anesthesia to ensure effective convulsions and calm awakening. Intraoperative vital signs were normal, with no signs of agitation post-treatment. Remimazolam administration for general anesthesia induction for mECT effectively induced convulsions and suppressed postoperative excitation. However, its effect on convulsions during mECT remains unclear, warranting further investigation.

## Introduction

Modified electroconvulsive therapy (mECT) is a technique that artificially induces seizures by administering electrical stimulations to the head under general anesthesia using short-acting sedatives and muscle relaxants. This is indicated for treating drug-resistant depression and schizophrenia. The primary general anesthetic agents include propofol and suxamethonium (succinylcholine chloride (SCC)). However, mECT is associated with immediate postoperative complications such as elevated blood pressure, agitation, and delirium [1,2]. Remimazolam, an ultrashort-acting benzodiazepine (BZ) intravenous anesthetic, has been recently approved in Japan for the induction and maintenance of general anesthesia. Notably, the incidence of circulatory depression was lower with remimazolam than with propofol [3]; further, remimazolam has an available antagonist. Herein, we report the case of postoperative agitation in a patient undergoing mECT with propofol who experienced effective seizure induction and mild arousal following sedation with remimazolam.

## Case presentation

**Figure 1:**
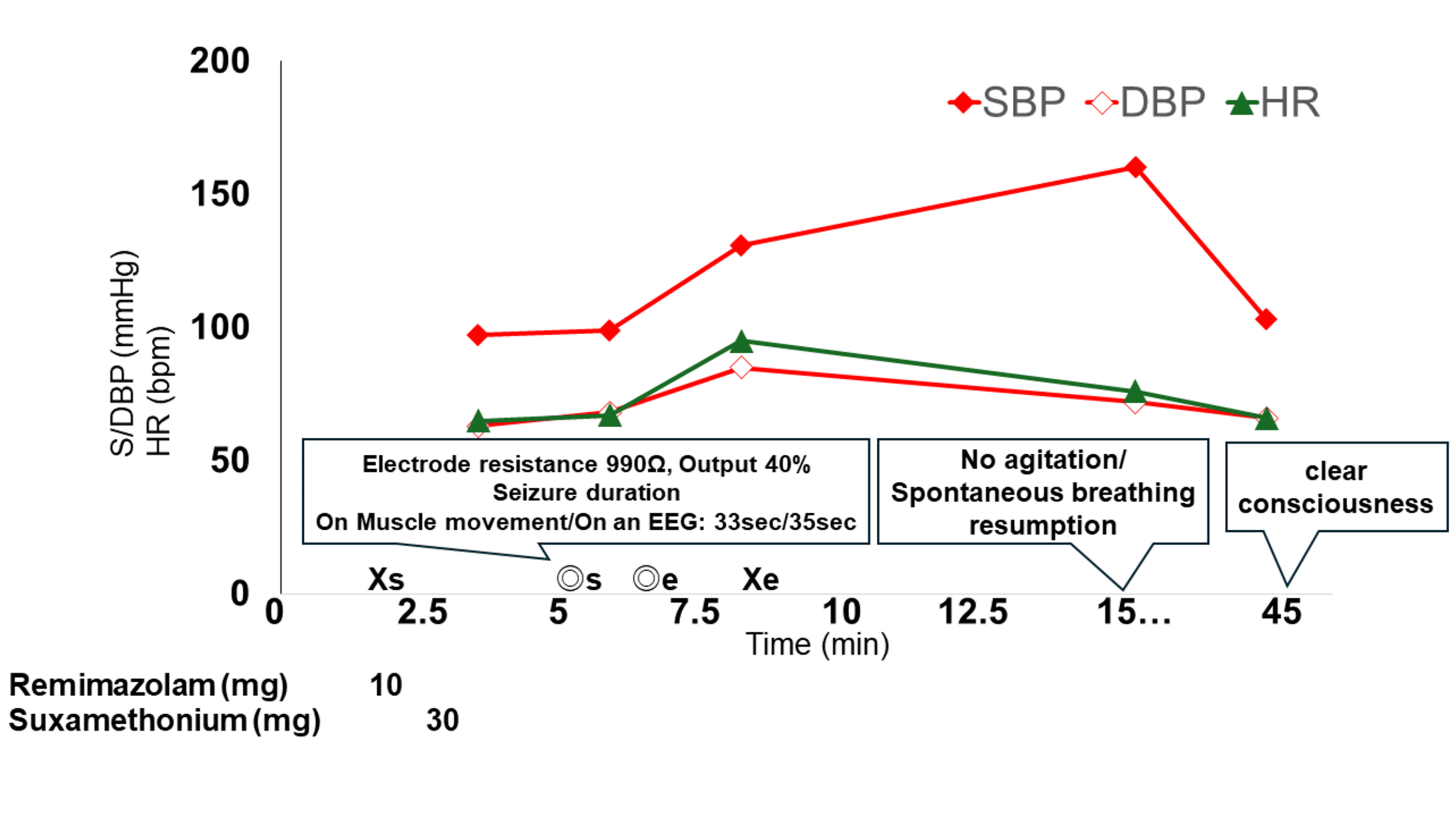
The anesthetic record DBP: diastolic blood pressure; EEG: electroencephalogram; HR: heart rate; SBP: systolic blood pressure; X: start and end of anesthesia; double circle: start and end of surgery

A 45-year-old man (height, 170 cm; weight, 52.7 kg) who was diagnosed with severe depression and psychotic symptoms was treated with vortioxetine hydrobromide, lemborexant, and risperidone. The patient had a history of dyslipidemia. The preoperative assessment revealed no abnormalities. The patient was treated with mECT. The first and second mECT sessions with propofol and SCC were uneventful. After the third treatment, the patient became agitated upon awakening, and 5 mg of diazepam was administered intravenously. However, owing to infusion leakage, 10 mg of midazolam was administered intramuscularly. Given the previous agitation episode, the fourth session of anesthesia induction with remimazolam was planned. Oxygen was administered at 6 L/min via a mask, and 10 mg (0.2 mg/kg) of remimazolam was administered intravenously. After confirming loss of consciousness, 30 mg of SCC was administered intravenously to induce muscle relaxation, and the patient was energized at 990 Ω of electrical resistance with 40% output. The muscle spasm duration was 33 seconds, and the electroencephalogram seizure duration was 35 seconds, indicating an effective seizure. The patient regained consciousness immediately after the procedure and resumed spontaneous breathing without signs of excitement. After returning to the room, the patient regained complete consciousness after 36 minutes and was able to speak. Vital signs remained stable during and after surgery (Figure 1).

Therefore, remimazolam was used for sedation until the seventh mECT session, consistently achieving mild arousal. Despite the absence of a reduction in seizure duration with the use of remimazolam, propofol was used for sedation for the subsequent eight consecutive sessions, taking into account the potential impact of BZ drugs on seizure duration; no complications were reported during these sessions. However, severe postoperative agitations were observed during the 15th mECT session, and 5 mg of diazepam was administered intravenously twice. From the 16th to the 24th sessions, mECT was performed using propofol, and no complications were observed.

## Discussion

Postoperative complications of mECT, including elevated blood pressure, agitation, and delirium, have been reported during the immediate postoperative period [1,2]. Furthermore, selectively energizing one side of the head in patients with a history of postoperative agitation reduced postoperative cognitive dysfunction [4], and preoperative administration of low-dose dexmedetomidine or midazolam was effective in managing postoperative agitation, resulting in seizures of appropriate durations and reduced recovery time [5]. Remimazolam is an ultrashort-acting BZ drug used for general anesthesia induction, similar to propofol [6]. Considering that remimazolam was reportedly more effective than propofol for managing postoperative delirium during transcatheter aortic valve replacement surgery [7] and hip replacement surgery [8], a suppressive effect on postoperative agitation was anticipated in our patient. The patient underwent 24 mECT sessions; remimazolam was used four times, and postoperative excitation was suppressed for all sessions. This may be explained by the fact that remimazolam has minimal effects on hemodynamics [6]. Hemodynamic instability, caused by increased fluctuations, has been identified as a predictor of postoperative delirium due to autonomic nervous system instability [9]. Additionally, a study reported that a greater heart rate variability during ECT increased the incidence of postoperative delirium [10]. While no reduction in the seizure duration was observed during these sessions, owing to the possibility that BZ drugs could shorten the seizure duration [11], the sedative was changed from remimazolam to propofol from the eighth session onward. Postoperative agitation observed again during the 15th session was managed with intravenous administration of diazepam (5 mg), suggesting that postoperative excitement may occur regardless of mECT treatment progression. Remimazolam can also be antagonized by flumazenil, potentially allowing safer sedation with mECT compared to other sedatives. The anterograde amnesic effects of remimazolam could be comparable to that of midazolam [12] and may be effective in treating post-traumatic stress disorder after mECT surgery.

Sedatives, such as thiopental, propofol, and midazolam, have been used in conjunction with mECT for medication-refractory depression and schizophrenia (Table 1).

**Table 1:** Characteristics of each sedative

	Thiopental	Propofol	Midazolam
Hemodynamic changes [1]	↑	↓	↓
Seizure duration [2,6]	+ ~ +++	++	+
Recovery time after a seizure [6]	++	+++	+
Characteristics	Some reports suggest that it has the longest seizure duration [6], while others suggest that it shortens it [2].	Blood pressure can be maintained at low levels [1], is short-acting [6], and has antiemetic effects. Hypertension and tachycardia have been observed in young patients [5].	Suppression of hemodynamic changes [6]. Can antagonize. Amnestic effects, headache, and muscle pain and cause nausea suppression [7].
Disadvantages	Increases heart rate and blood pressure, elicits gag reflex, induces cough, and provokes tearing. Causes delayed awakening from anesthesia, irregular pulse, glottic spasm, and postoperative nausea and vomiting [6]	Shortening of seizure duration [6]; pain on injection [6]	Shortening of seizure duration [6]; longer half-life
Use frequency	Not often used [2]	Most used [2]	Not often used [2]

Thiopental is not currently used because of disagreements regarding seizure duration and the high incidence of adverse events (e.g., cardiovascular effects and frequent side effects). Although propofol causes dose-dependent hypotension and vasorelaxation, it is most frequently used because of its short half-life and minimal impact on seizure duration [1]. Despite the minimal hemodynamic effects, availability of antagonists, and amnesic properties, midazolam is avoided because of its tendency to shorten seizure duration [11,13]. Given that remimazolam is a BZ, there was concern regarding its potential to shorten seizure duration; however, effective seizures were obtained in our patient. Although remimazolam and midazolam have almost equivalent hypnotic potencies, remimazolam has an ester bond in its diazepine ring, which allows it to be rapidly hydrolyzed by tissue esterases. Also, remimazolam's metabolite has an extremely low affinity for BZ receptors: about 1/400 of that of midazolam [14]. These differences explain the short lifespan of remimazolam's effects. This may explain why, unlike other BZs, remimazolam does not shorten the duration of seizure during ECT. Studies examining the success rate of remimazolam induction with a single intravenous dose of 0.2 mg/kg, 0.3 mg/kg, and 0.4 mg/kg reported no significant difference than did propofol at doses of ≥0.3 mg/kg [15]. In mECT, lower doses of propofol have less effects on seizure duration [16]. Therefore, considering the effect on convulsions, a lower dose of remimazolam (0.2 mg/kg) was administered to our patient. A study that investigated the achievement of reliable loss of consciousness during induction of general anesthesia across different age groups - young (20-39 years), middle-aged (40-59 years), and elderly (60-79 years) - using bolus administration of remimazolam reported that the remimazolam dose required to achieve the loss of consciousness in 95% of the population was 0.367, 0.369, and 0.249 mg/kg in the young, middle-aged, and elderly groups, respectively [17]. Since the patient, in this case, falls into the middle-aged category, it is possible that 0.369 mg/kg of remimazolam was required to achieve reliable loss of consciousness. The insufficient dose of remimazolam may have also contributed to the failure to shorten the seizure duration. The dosage could have been increased if sleep onset was not achieved; however, successful loss of consciousness was achieved without the need for further dosage adjustment. The effect of remimazolam on mECT convulsions remains unclear and necessitates further validation.

## Conclusions

Remimazolam administration in a patient with a history of postoperative agitation after mECT was effective in achieving adequate convulsions and suppressing postoperative excitation. Remimazolam, whose antagonist is available, offers advantages over propofol in terms of ease of adjusting sedation. However, its overall impact on mECT-induced convulsions remains unclear. Further studies are warranted to elucidate the impact of remimazolam on mECT.
